# Enhanced polyhydroxyalkanoate (PHA) production from the organic fraction of municipal solid waste by using mixed microbial culture

**DOI:** 10.1186/s13068-017-0888-8

**Published:** 2017-08-22

**Authors:** Bianca Colombo, Francesca Favini, Barbara Scaglia, Tommy Pepè Sciarria, Giuliana D’Imporzano, Michele Pognani, Anna Alekseeva, Giorgio Eisele, Cesare Cosentino, Fabrizio Adani

**Affiliations:** 1 Gruppo Ricicla labs-DiSAA-Università degli Studi di Milano, Via Celoria 2, 20133 Milan, Italy; 2 Centro Alta Tecnologia Istituto di Ricerche Chimiche e Biochimiche G. Ronzoni Srl, Via Colombo 81, 20133 Milan, Italy; 3 Istituto di Ricerche Chimiche e Biochimiche G. Ronzoni, Via Colombo 81, 20133 Milan, Italy

**Keywords:** Aerobic dynamic feeding strategy, Anaerobic percolation biocell reactor, Mixed microbial culture, Municipal solid waste, Polyhydroxyalkanoate, Sequencing batch reactor

## Abstract

**Background:**

In Europe, almost 87.6 million tonnes of food waste are produced. Despite the high biological value of food waste, traditional management solutions do not consider it as a precious resource. Many studies have reported the use of food waste for the production of high added value molecules. Polyhydroxyalkanoates (PHAs) represent a class of interesting bio-polyesters accumulated by different bacterial cells, and has been proposed for production from the organic fraction of municipal solid waste (OFMSW). Nevertheless, until now, no attention has been paid to the entire biological process leading to the transformation of food waste to organic acids (OA) and then to PHA, getting high PHA yield per food waste unit. In particular, the acid-generating process needs to be optimized, maximizing OA production from OFMSW. To do so, a pilot-scale Anaerobic Percolation Biocell Reactor (100 L in volume) was used to produce an OA-rich percolate from OFMSW which was used subsequently to produce PHA.

**Results:**

The optimized acidogenic process resulted in an OA production of 151 g kg^−1^ from fresh OFMSW. The subsequent optimization of PHA production from OA gave a PHA production, on average, of 223 ± 28 g kg^−1^ total OA fed. Total mass balance indicated, for the best case studied, a PHA production per OFMSW weight unit of 33.22 ± 4.2 g kg^−1^ from fresh OFMSW, corresponding to 114.4 ± 14.5 g kg^−1^ of total solids from OFMSW. PHA composition revealed a hydroxybutyrate/hydroxyvalerate (%) ratio of 53/47 and Mw of 8∙10^5^ kDa with a low polydispersity index, i.e. 1.4.

**Conclusions:**

This work showed how by optimizing acidic fermentation it could be possible to get a large amount of OA from OFMSW to be then transformed into PHA. This step is important as it greatly affects the total final PHA yield. Data obtained in this work can be useful as the starting point for considering the economic feasibility of PHA production from OFMSW by using mixed culture.

**Electronic supplementary material:**

The online version of this article (doi:10.1186/s13068-017-0888-8) contains supplementary material, which is available to authorized users.

## Background

Food waste (FW) is defined as the organic material produced for human consumption and discarded, lost or degraded, primarily at manufacturing, retail and consumption stages [1]. In Europe, almost 87.6 million tonnes of FW are produced annually [2]. These wastes are characterized by both high moisture and high biodegradability, so FW creates adverse environmental impacts if landfilled (odours, fires, volatile organic compounds, groundwater contamination by leachate, global climate changes, etc.) and also, high disposal costs (88–144 $ tonne^−1^) [1]. European and national legislation have focused on avoiding FW landfilling by treating it through a thermal process (incineration) or, more frequently, by biological processes (anaerobic digestion and composting) to be carried out on the separately collected organic fraction of municipal solid waste (OFMSW) [3]. Despite the high biological value of FW, traditional management solutions do not consider it as a precious resource, adding only a small amount of value to the final product, i.e. 60–150 $ tonne^−1^ of biomass for electricity production and 5–10 $ tonne^−1^ of biomass for compost production [4]. Therefore, supporting these activities generally requires government contributions or high tariffs for citizens, e.g. in Italy the waste tariff increased by 70% in the last 10 years.

FW is an organic matrix rich in valuable molecules such as starch, cellulose, hemicellulose, lignin, proteins, lipids and organic acids that could be managed in a more sustainably economic way by using it as a raw material for bulk chemicals production [4]. Many studies have reported the use of FW for the production of high added value molecules such as lactic acid, citric acid, succinic acid, single cell oils, enzymes and polymers [5], an approach which is more financially rewarding in comparison with compost and biomethane production (1000 $ tonne^−1^ of biomass for bulk chemicals production from FW) [6].

Among the wide varieties of bio-products obtainable from FW are the polyhydroxyalkanoates (PHAs), a class of interesting bio-polyesters accumulated by different bacterial cells under the form of granules inside the cytoplasm. PHAs are completely biodegradable and are mainly produced starting from renewable sources; their chief interesting property is their mechanical behaviour that make them comparable to common plastics [7]. Microbial PHA production can be carried out by using either pure or mixed microbial cultures (MMC). MMC with high PHA accumulation capacity have been suggested as a solution for reducing the high maintenance costs of pure cultures. Many studies related to PHA production by MMC have performed both selection of PHA-storing cultures and PHA accumulation by using just OA as substrates, since they are the direct metabolic precursors of PHA [7], while PHA production carried out by using pure cultures allows the employment of both OA and simple sugars as carbon sources. Working with mixed microbial cultures, simple sugars are not recommended as they can be used by the culture for the production of other molecules (i.e. glycogen), reducing the final PHA production yield [8, 9]. Given the high costs of synthetic OA, agro-industrial wastes, such as molasses, cheese whey, olive oil mill effluent, palm oil mill effluent, candy bar factory wastewater and many other waste streams [10–14], can be treated by acidic fermentation to produce OA which can then be transformed into PHA by MMC.

Complex wastes, such as OFMSW, have been reported in the literature to be used as substrates for PHA production by MMC [15–18]. These studies focused attention on PHA production from OA obtained by the fermentation of OFMSW, but until now, no attention has been paid to the entire process leading to the transformation of OFMSW to OA and then to PHA, i.e. to obtain overall estimates of PHA yield based on OFMSW weight unit used. These data, together with those showing the scientific feasibility in transforming organic waste into PHA, are important to estimate the effective economic sustainability of PHA production from OFMSW.

In this work, a two-stage approach, i.e. OA production from OFMSW and subsequent PHA biosynthesis from OA, is proposed.

Optimization of the acidic fermentation of OFMSW was carried out in order to exploit the biological potential of this complex matrix, obtaining a percolate rich in OA used subsequently as the substrate to produce PHA by employing MMC. Global mass balance of the process performed was achieved as well as the complete characterization of the polymer obtained.

## Methods

### Percolates production from OFMSW

#### Organic fraction of municipal solid waste collection

The source-separated OFMSW coming from street bin containers was collected at a full-scale composting plant located in northern Italy. Collection was done by following the quartering method: in brief, the OFMSW was sampled at different points of the whole mass (500 kg wet weight − w.w. − ×3 times); then the collected waste was mixed, quartered and sub-sampled until a final sample of about 300 kg w.w. material was reached. OFMSW was stored at 4 °C before the trials were set up. A representative sample of approximately 30 kg, obtained by mixing sub-samples of about 5 kg each taken randomly from sampled material, was dried and crushed to 2 mm and then used to perform analytical analyses.

The bulking agent (shredded wood) from the same plant was also collected following a similar approach.

Liquid digestate, used to irrigate OFMSW during dry anaerobic fermentation (see later), was collected directly from a continuously stirred tank reactor anaerobic digester (CSTR-AD) plant fed with OFMSW, located in northern Italy and previously described [19]. Digestate was collected from this plant in large quantities from the discharge pipe system of the post-digester after 30 days of retention time (HRT).

#### Experimental apparatus

**Fig. 1:**
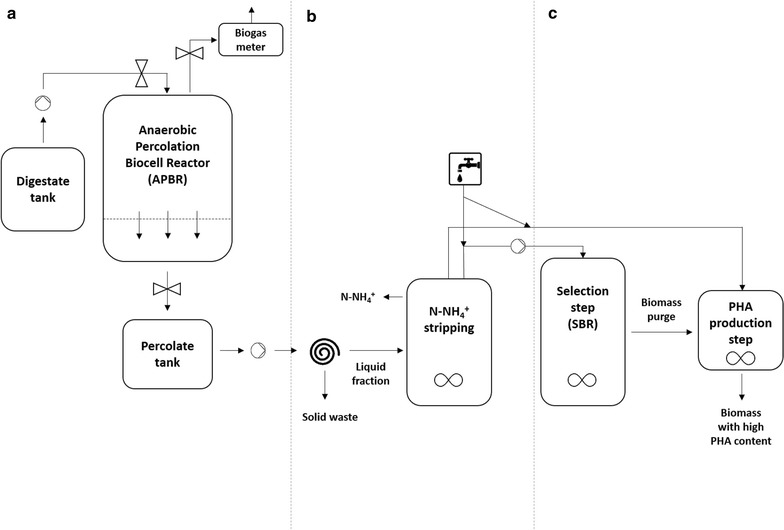
Scheme of the two-stage process used to produce PHA: OFMSW fermentation producing OA (**a**), percolate pre-treatments (**b**) and PHA production (**c**)

The acidogenesis (SSAD) trials were carried out by developing a pilot-scale anaerobic percolation biocell reactor (APBR) that was made up of an insulated vertical cylinder (100 L of volume) of PVC material with a hermetic cap. Inside the reactor, there was a stainless steel basket with holes at the bottom, into which OFMSW was introduced from the APBR cap, as well as the digestate used to irrigate the organic wastes (Fig. 1). From the bottom of the APBR, the percolate generated daily due to digestate irrigation was extracted and weighed. Digestate irrigation was used to buffer the acidity produced during OFMSW fermentation and to remove OA produced in the APBR. This encouraged high hydrolysis performance from the organic waste, which is the limiting step in producing large amounts of OA from organic substrates [20, 21]. Moreover, large-scale OA production becomes toxic for methanogenic bacteria, limiting methane production during the trials.

#### Experimental setup

Three different percolation trials were carried out. For all of them, fresh OFMSW (85% w.w.) and bulking agent (chopped green waste material) (15% w.w.) were mixed just before reactor filling to avoid clogging problems in the APBR, allowing the percolation. For each trial, the APBR was filled with about 50 kg of mixture (OFMSWmix-in).

The OFMSWmix-in in the APBR was irrigated continuously by using the liquid digestate obtained from the full-scale CSTR-AD plant in order to optimize OFMSW hydrolysis and subsequent acidogenesis fermentation to produce OA. Anaerobic hydrolysis and acidic fermentation lasted for 21 days for each trial performed. During this time, irrigation with the liquid digestate (previously heated to 55 °C) was done continuously by using a peristaltic pump, adopting an OFMSWmix-in/digestate ratio of 1:0.45 kg kg^−1^ (flow rate of 0.02 L h^−1^ kg^−1^ OFMSW_w.w._) for the first trial (Trial 1), 1:0.9 kg kg^−1^ (flow rate of 0.04 L h^−1^ kg^−1^ OFMSW_w.w._) for the second trial (Trial 2), and of 1:1.8 kg kg^−1^ (flow rate of 0.08 L h^−1^ kg^−1^ OFMSW_w.w._) (day 1–8) and of 1:0.9 kg kg^−1^ (flow rate of 0.04 L h^−1^ kg^−1^ OFMSW_w.w._) (day 9–21) for the third trial (Trial 3), respectively. The daily-generated percolates were collected from the bottom of the APBR, quantified and mixed together to get a final mixed percolate sample for each trial.

### Percolates treatment before PHA production

The three percolates obtained from anaerobic acidic fermentation trials were submitted to two pre-treatments before their use for polyhydroxyalkanoate (PHA) production. The first pre-treatment was to centrifuge the percolates at 20,000*g* for 15 min, reducing suspended organic carbon from percolates. Supernatants obtained underwent a second pre-treatment adopted to remove ammonia by stripping. This step was performed by bringing supernatant-pH to 11 by adding 6-mol L^−1^ KOH and stirring it (magnetic stirring at 200 rpm) under the hood until a final C:N close to 10 was reached [11]. Then ammonia stripping was stopped and the pH was brought again to the initial value of 8 by using 3 mol L^−1^ H_2_SO_4_. Ammonia stripped was not trapped at lab scale and it was not considered in this work. Obviously, this step needs to be considered and further studied on a larger scale.

After pre-treatments, the three supernatants were diluted with deionized water, to produce a final COD content close to 1300 mg L^−1^ [11]. These OFMSW-derived supernatants (OFMSW-supernatants_in_) were used for subsequent inoculum selection.

Substrates (OFMSW-supernatants_acc._) used for PHA accumulation tests were derived from percolates that had undergone the same treatments shown for those destined for the inoculum selection. Three OFMSW-supernatants_acc._ were obtained but, in this case, the ammonia content was lower than those reported for OFMSW-supernatants_in_, since it has been reported that N starvation can determine a greater conversion of carbon into PHA because of cell growth limitation [22]. Moreover, OFMSW-supernatants_acc._ were less diluted, giving a final COD concentration of about 7500 mg L^−1^, in order to avoid an excessive dilution of the biomass in the accumulation reactor [10].

### Inoculum production and PHA-producing bacteria enrichment

The enrichment of PHA-producing bacteria was performed by using an inoculum constituted by an activated sludge (5 g total suspended solids L^−1^) collected from the secondary sedimentation tank of a wastewater treatment plant (5.2 × 10^5^ equivalent inhabitants) located at Peschiera Borromeo (Milan, Italy).

The enrichment in PHA-producing bacteria was carried out in a Sequencing Batch Reactor (SBR) with a working volume of 800 mL, applying an aerobic dynamic feeding (ADF) strategy [11]. In brief, the SBR cycle length was of 12 h, consisting of four discrete phases: (1) influent filling (4 min), (2) aeration (675 min), (3) settling (40 min) and (4) withdrawal of the exhausted effluent (5 min), with 1 day of hydraulic retention time (HRT) and 5 days of Sludge Retention Time (SRT), keeping the temperature at 25 °C and the pH at 8.8, this latter controlled automatically by adding 1 mol L^−1^ HCl. Aeration and agitation were provided by supplying air at 6 L min^−1^ and stirring set at 110 rpm. Pumping, aeration and stirring were automatically controlled.

The selection process lasted for 3 months; for each month, a different pre-treated supernatant was used as substrate: OFMSW-supernatant_in_ 1, OFMSW-supernatant_in_ 2 and OFMSW-supernatant_in_ 3, respectively, for the first, second and third month of selection.

The selection trend was monitored by determining the duration of the feast phase achievable by using the dissolved oxygen (DO) concentration in the selection media [10], measured by an optical probe (FDO 925, WTW, Germany).

In particular, the feast (h)-to-famine (h) ratio (F/F ratio) was calculated as the ratio between the lengths in hours of the two phases. For a correct selection of PHA-storing bacteria, the F/F ratio had to be equal or less than 0.33 [23]. To carry out the selection of PHA-accumulating bacteria, 400 mL of activated sludge was used as the inoculum feed for each cycle, with 400 mL of OFMSW-supernatant_in_. Organic Loading Rate (OLR) was kept close to 1300 mg COD L^−1^ day^−1^ and the C:N:P ratio was of about 100:13:0.4 mol C: mmole N: mmole P. Every month, two cycles were monitored in order to evaluate the performance of the selected culture. In particular, monitoring was performed between cycles 31 and cycle 45 since it was reported that the cultures reach stability after three SRTs from the beginning of the trial (cycle 31) [24].

### PHA accumulation

The ability of the MMC to accumulate PHA was assessed by fed-batch assays carried out in a 300-mL working volume glass reactor, with continuous aeration and stirring. These assays consisted of feeding the substrate to 160 mL of enriched culture (at least 3 SRTs from the beginning of the selection) [24] adopting a pulse-wise feeding method. The assays were monitored continuously by measuring the concentration of the dissolved oxygen in the accumulation media [10]. In particular, substrate (OFMSW-supernatant_acc._) was fed to the reactor when DO showed a strong increase [10]. Total C dosed was calculated taking into account that the ratio of the carbon to the microorganisms had to be the same as that inside the selection reactor. The assays were stopped when no DO variation followed the substrate feeding.

For the accumulation tests, the operating conditions used were those adopted in the selection reactor, i.e. temperature of 25 °C, aeration of 6 L min^−1^ and stirring at 110 rpm.

The biomass from the selection process was submitted to accumulation tests using the same substrate as the carbon source; every month, two accumulation trials were performed in duplicate, between the cycles 31 and 45.

### PHA extraction

The biomass collected after PHA accumulation tests was centrifuged at 8000*g* for 15 min, washed with 0.9% sodium chloride solution and centrifuged again at 8000*g* for 20 min. The pellet obtained was lyophilized and then suspended in chloroform (in a ratio of ca. 40 mL CHCl_3_ g^−1^ dried cells) and left to dissolve for a period of 3 days at 37 °C [10]. The solution was then filtered to remove all undissolved material. The extracted PHA in chloroform was then precipitated by the addition of 5 volumes of methanol and allowed to settle down for 30 min [25]. The white precipitate formed was then filtered, suspended in chloroform and used to fill glass Petri dishes. Finally, chloroform was evaporated allowing polymer recovery in the form of a thin film.

### Analytical procedures

#### OFMSW, digestate and percolates characterization

Total solids (TS), volatile solids (VS), total nitrogen (TKN), pH and ammonia (N-NH_4_^+^) (detected on fresh material) contents were determined according to the standard procedures [26]. Total OA expressed as acetic acid, were detected on fresh material and determined according to the acid titration method [27].

#### Substrate and biomass characterization during PHA production

The substrates (OFMSW-supernatants) fed during the selection and accumulation processes were characterized in terms of pH, TS, VS, Chemical Oxygen Demand (COD), OA content (acetate, butyrate, lactate, propionate and valerate), TKN, N-NH_4_^+^ and phosphorus (P) content.

During the selection trials, samples were taken during the cycle once in each SRT; every sample was characterized in terms of total suspended solids (TSS), volatile suspended solids (VSS), soluble COD, OA content, N-NH_4_^+^ content and PHA content. During accumulation trials, samples were taken continuously in order to measure TSS, VSS, soluble COD, OA content and PHA content. Biomass concentration was calculated as VSS according to the standard methods [10].

TSS and VSS were determined as reported by Valentino et al. in 2015 [28]. OA concentrations measured on filtered samples (filter diameter of 0.45 µm) were determined by high-performance liquid chromatography (HPLC) using a chromatograph equipped with a UV detector and Aminex HPX-87H column (column temperature 20 °C, 0.0025 M H_2_SO_4_ eluent, flow rate 0.6 mL min^−1^). The OA concentrations were calculated through a standard calibration curve (20–1000 mg L^−1^ of each organic acid). The COD and the N-NH_4_^+^ content (filtered at 0.45 µm) were determined using cuvette test kits (Macherey-Nagel, Germany).

PHAs were determined by GC MS using a method adapted from Serafim et al. in 2004 [22]. Lyophilized biomass was incubated for methanolysis in a 20% v/v H_2_SO_4_ in MeOH solution (1 mL) and extracted with chloroform (1 mL). The mixture was digested at 100 °C for 3.5 h. After the digestion step, the organic phase (methylated monomers dissolved in chloroform) was extracted and injected (1 mL) into a gas chromatograph equipped with a detector (7980, Agilent Technologies, USA) and a ZB-Wax column (30 m, 0.25 mm internal diameter, 0.25 µm film thickness, Zebron, Phenomenex, USA), using helium as a carrier gas at 1.0 mL min^−1^. Samples were analysed under a temperature regime starting at 40 °C, increasing to 100 °C at a rate of 20 °C min^−1^, to 175 °C at a rate of 38 °C min^−1^ and reaching a final temperature of 220 °C at a rate of 20 °C min^−1^ for ensuring cleaning of the column after each injection. Injector and detector temperatures were at 280 and 230 °C, respectively. Hydroxybutyrate (HB) and hydroxyvalerate (HV) concentrations were determined through the use of two calibration curves, one for HB and another for HV, using standards (0.1–8 g L^−1^) of a commercial P (HB–HV) (88/12%) (Sigma-Aldrich, Germany), and corrected using heptadecane as internal standard (concentration of approximately 1 g L^−1^) (Sigma Aldrich, Germany).

#### PHA and active biomass growth yield calculation

The PHA content in cells was referred to VSS on a mass basis [PHA = (g kg^−1^ VSS)], considering VSS to be constituted by both active biomass (X) and PHA [10]. PHA was converted into COD according to the following oxidation stoichiometry: 1.67 mg COD mg^−1^ HB monomer and 1.92 mg COD mg^−1^ HV monomer [23].

Acetate, butyrate and lactate were considered as HB precursors, valerate and propionate as HV precursors [10]. X was calculated on a COD basis considering that 1 g of X contains 1.42 g of COD [23]. For the SBR, PHA storage yield was expressed in COD and referred to both COD consumed (COD_cons._) and OA consumed (COD_OA-cons._), calculated, respectively, as the ratio between the amount of PHA accumulated during the feast phase (COD_PHA_) and the amount of COD depleted or OA depleted.

In the accumulation batches, these PHA storage yields were calculated as described before, for each pulse. In order to compare different accumulation tests, the average values of the first three pulses and for each parameter were considered [23].

In accumulation batches, PHA storage yield was also related to COD fed (COD_in_) and OA fed (COD_OAin_); moreover, it was also related back to total solids of OFMSW (OFMSW_TS_) and to OFMSW_w.w._.

The X growth yield in the SBR was expressed in COD and referred to COD consumed (COD_cons._), calculated as the ratio between the new X produced during the feast phase (COD_X_) and the amount of COD depleted [23].

#### PHA characterization by solid-state NMR and solution ^1^H NMR, and molecular weight

Solid-state ^13^C NMR spectra of lyophilized biomass containing PHA were recorded on a Bruker Avance 300 spectrometer operating at 75.47 MHz, using a 4 × 21 mm cylindrical zirconium rotor spun at 11,000 Hz to avoid the side bands. The ^13^C cross-polarization magic angle spinning (CPMAS) NMR spectra were acquired using recycle delay of 8 s, ^1^H 90 pulse length of 3.5 μs, 1 m contact time, acquisition time of 35 ms and from 1K to 4K scans. The ^13^C single-pulse excitation (SPE) NMR spectra were recorded with delays of 160 s and 1–2K scans.

The chemical shifts were recorded relative to tetramethylsilane via benzene as a secondary reference.

Liquid NMR experiments on extracted PHA were performed on a Bruker 500 MHz AVANCE III NMR spectrometer (Bruker GmbH, Germany) with a 5-mm TCI cryoprobe. Deuterated chloroform (99.96%, Sigma Aldrich) was used as solvent. ^1^H NMR spectra were recorded at 303 K using recycle delay of 12 s, 64K fid size and 64 scans.

PHA molecular weight was determined by HP-SEC/TDA measurement. The HPLC equipment consisted of a Viscotek system (Malvern Instrument Ltd, Malvern, UK) equipped with a Knauer HPLC pump K501, and a Biotech Degasi GPC degassing device. The detector system was a Viscotek mod. 302 Triple Detector Array (TDA), which is composed by Laser Light Scattering detector (90° and 7°; wavelength 670 nm), Refractive index (RI) detector [cell volume of 12 μL; light-emitting diode (LED) at 660 nm wavelength] and Viscosimeter detector (four capillaries with a differential Wheatstone bridge configuration). A PL GEL 20-um MIXED A column (7.5 × 300 mm) was used. Chloroform was used as the mobile phase at a flow rate of 1 mL min^−1^. Columns, injector and detectors were maintained at 30 °C. Samples were dissolved in chloroform at concentrations of 2–8 mg mL^−1^ and filtered on a 0.2-μm membrane before injection. Injection volume was of 100 μL.

The system was calibrated with the PS narrow standard of known Mw, polydispersity and intrinsic viscosity (Malvern PolyCAL PS std 105 k). Using a standard PHA sample at different concentrations (2.3, 4.3, 6.0, 8.5 mg mL^−1^), the differential refractive index increment (dn/dc) value was found to be equal to 0.024 and used for further calculations.

#### Statistical analysis

Average and standard deviation values were calculated according to standard procedures and the results were analysed by an ANOVA test. A Tukey’s test was used to compare mean values and to assess the significance of the differences between mean values, adopting a fixed effects model, i.e. the digestate flow adopted modified the chemical composition of both the percolates produced and the supernatants derived.

All statistical analyses were carried out using the SPSS statistical software, version 15.0 (SPSS, Chicago, IL, USA).

## Results

### Percolation trials

Percolation trials carried out by adopting different irrigation regimes, i.e. varying digestate flow rate, showed differences in terms of total percolate produced, i.e. an increase of the hourly digestate flow rate determined an increase of the total percolate produced (Table 1). Again, the production of total OA increased by more than 200% when digestate flow rate doubled, when comparing Trial 1 with Trial 2 (Table 1). However, the further increase of digestate flow rate (Trial 3) did not lead to any increase of OA production and on the contrary, slightly decreased it.

**Table 1:** Percolation trials conditions (a) and composition of OFMSWs and digestates used and of percolates produced (b)

Trial	T (°C)	Duration (day)	OFMSW (kg)	Digestate flow rate (L h^−1^ kg^−1^ OFMSW_w.w.)_	Percolate produced (L kg^−1^ OFMSW_w.w._)	OA produced^a^ (g kg^−1^ OFMSW_w.w._)
a						
Trial 1	30	21	44	0.02	8.03	60.5
Trial 2	30	21	42.7	0.04	19.3	151
Trial 3	40 (day 1–8) 30 (day 9–21)	21	43.6	0.08 (1–8 day) 0.04 (9–21 day)	25.57	139

Trial	TS (%)	VS (%)	pH	OA^a^ (mg L^−1^)	N-NH_4_^+^ (mg L^−1^)
b					
Trial 1, 2, 3					
OFMSW (average)	24.5 ± 3.9	86.8 ± 2.6	4.3 ± 0	11,663 ± 1.649	304 ± 239
Trial 1, 2, 3					
Digestate (average)	2.2 ± 0.2	70.8 ± 2.7	8.32 ± 0.1	3448 ± 1.591	2065 ± 343
Trial 1					
Percolate 1 (P1)	2.4 ± 0.49a^b^	71.2 ± 2.2b	6.91 ± 0.19a	7540 ± 1,474b	2231 ± 130b
Trial 2					
Percolate 2 (P2)	1.9 ± 1.1a	66.5 ± 2.8a	6.99 ± 0.61a	7831 ± 1,775b	2412 ± 472b
Trial 3					
Percolate 3 (P3)	2 ± 0a	73 ± 2a	6.99 ± 0.32a	5437 ± 818a	1784 ± 117a

^a^OA expressed as acetic acid, determined according to the acid titration method [27]

^b^Limited to percolates, values in the same column followed by the same letter are not statistically different (ANOVA, Tukey test, *p* < 0.05)

Percolates obtained were similar in terms of chemical characteristics apart from the percolate coming from the third trial that appeared more diluted as regarded OA and N-NH_4_^+^ content (Table 1).

Trial 2 optimized OFMSW hydrolysis and subsequent acidogenesis fermentation, producing the highest amount of OA.

### OFMSW-supernatants

Pre-treated percolates used for MMC production (OFMSW-supernatants_in_) showed no statistical differences in terms of COD and N-NH_4_^+^ contents, apart from the P content and HB/HV precursors ratio which were higher for OFMSW-supernatant_in_ 3 than for the other OFMSW-supernatants_in_ (Table 2).

**Table 2:** Chemical composition of the substrates used in the MMC selection and PHA accumulation processes

Substrate	COD (mg L^−1^)	N-NH_4_^+^ (mg L^−1^)	P tot (mg L^−1^)	C (mmol L^−1^)	N-NH_4_^+^ (mmol L^−1^)	P (mmol L^−1^)	Organic acids composition (%, weight basis, HB precursors/HV precursors)
OFMSW-supernatant_in_ 1	1304 ± 10a^a^	46.7 ± 4.9a	3.1 ± 0.3a	34.4 ± 0.3a	2.6 ± 0.3a	0.1 ± 0a	59.7/40.3
OFMSW-supernatant_in_ 2	1322 ± 37°	56.2 ± 7.5a	3.6 ± 0.4a	34.9 ± 1a	3.1 ± 0.4a	0.12 ± 0.01a	51.2/48.8
OFMSW-supernatant_in_ 3	1231 ± 63a	58.4 ± 8.1a	5.6 ± 0.6b	32.5 ± 1.7a	3.2 ± 0.5a	0.18 ± 0.02b	67.8/32.2
OFMSW-supernatant_acc._ 1	7591 ± 54a	28 ± 4a	16.9 ± 2.3a	200 ± 2a	1.6 ± 0.2a	0.55 ± 0.07a	57.2/42.8
OFMSW-supernatant_acc._ 2	7879 ± 87a	29.1 ± 2a	19.8 ± 3a	207.8 ± 2.3a	1.6 ± 0.1a	0.64 ± 0.11a	52/48
OFMSW-supernatant_acc._ 3	7253 ± 98a	26.8 ± 3a	31.1 ± 5.2b	191.3 ± 2.6a	1.5 ± 0.2a	1 ± 0b	65.1/34.9

^a^By separating OFMSW-supernatants_in_ from OFMSW-supernatants_acc._, values in the same column followed by the same letter are not statistically different (ANOVA, Tukey test, *p* < 0.05)

OFMSW-supernatants used to perform PHA accumulation tests (OFMSW-supernatants_acc._) were again quite similar to each other, with the exception for P content and HB/HV precursors ratio which were higher for OFMSW-supernatant_acc._ 3 than for the other OFMSW-supernatants_acc._ (Table 2).

### Mixed microbial culture enrichment

The amount of PHA stored at the end of the feast phase and PHA storage yields, measured for the mixed microbial culture during the selection process, remained quite stable (Table 3) during the first 2 months of selection when OFMSW-supernatants_in_ 1 and 2 were used as substrates. In particular, PHA stored at the end of the feast phase was subject to small fluctuations with an average value of 363 ± 30 g kg^−1^ VSS. PHA storage yields (expressed as COD) on COD and on OA consumed were, on average, of 0.52 ± 0.07 mg mg^−1^ COD_cons._, and of 0.86 ± 0.23 mg mg^−1^ COD_OA-cons._, respectively, indicating the main role of OA among different carbon sources in PHA production [7, 11].

**Table 3:** Parameters characterizing the MMC selection and PHA accumulation processes with the three OFMSW-supernatants

Substrate	PHA content^a^ (g kg^−1^ VSS)	Polymer composition (ΔHB/ΔHV) (% w/w)	PHA yield^c^ (mg mg^−1^ COD_cons._)	PHA yield^d^ (mg mg^−1^ COD_OA-cons._)	PHA yield^e^ (g kg^−1^ COD_in_)	PHA yield^f^ (g kg^−1^ OA_in_)	PHA productivity (g L^−1^ day^−1^)	X growth yield^g^ (mg mg^−1^ COD_cons._)
*Selection*								
OFMSW-supernatant_in_ 1	342 ± 23	–	0.51 ± 0.02	0.85 ± 0.26	–	–	–	0.16 ± 0.01
OFMSW-supernatant_in_ 2	383 ± 2	–	0.54 ± 0.07	0.87 ± 0.09	–	–	–	0.05 ± 0.01
OFMSW-supernatant_in_ 3	234 ± 20	–	0.42 ± 0.04	1.15 ± 0.34	–	–	–	0.09 ± 0.01
*Accumulation*								
OFMSW-supernatant_acc._ 1	476 ± 41^b^	56.5 ± 3.5/43.5 ± 3.5	0.44 ± 0.17	1.13 ± 0.18	134 ± 22	230 ± 38	4.4 ± 1.3	–
OFMSW-supernatant_acc._ 2	465 ± 21^b^	54.5 ± 2.6/45.5 ± 2.6	0.52 ± 0.1	0.75 ± 0.21	128 ± 16	220 ± 28	4.3 ± 1.2	–
OFMSW-supernatant_acc._ 3	405 ± 28^b^	57 ± 2/43 ± 2	0.5 ± 0.1	0.66 ± 0.09	94 ± 5	161 ± 9	7 ± 0.9	–

^a^PHA stored at the end of the feast phase referred to VSS

^b^PHA accumulated at the end of the accumulation test referred to VSS

^c^PHA storage yield expressed as COD (COD_PHA_) referred to COD_cons._

^d^PHA storage yield expressed as COD (COD_PHA_) referred to COD_OA-cons._

^e^PHA produced referred to COD_in_

^f^PHA produced referred to OA_in_

^g^X growth yield during feast phase expressed as COD (COD_X_) referred to COD_cons._

The culture fed with OFMSW-supernatant_in_ 3 gave different results as the polymer content at the end of the feast phase and PHA storage yield on COD consumed were lower by 36 and 20% in comparison with the data reported for OFMSW-supernatants_in_ 1 and 2. On the other hand, PHA storage yield on OA consumed increased by 34%. In any case, performance obtained with OFMSW-supernatant_in_ 3 was better than that reported in the literature for similar substrates (OFMSW leachate) [17, 18] and it was comparable to those obtained with other waste substrates (e.g. fermented molasses and fermented cheese whey) [10, 29]. Probably, differences found in terms of reduction of polymer content were because OFMSW-supernatant_in_ 3 was characterized by a higher presence of P that reduced PHA accumulation [30].

**Fig. 2:**
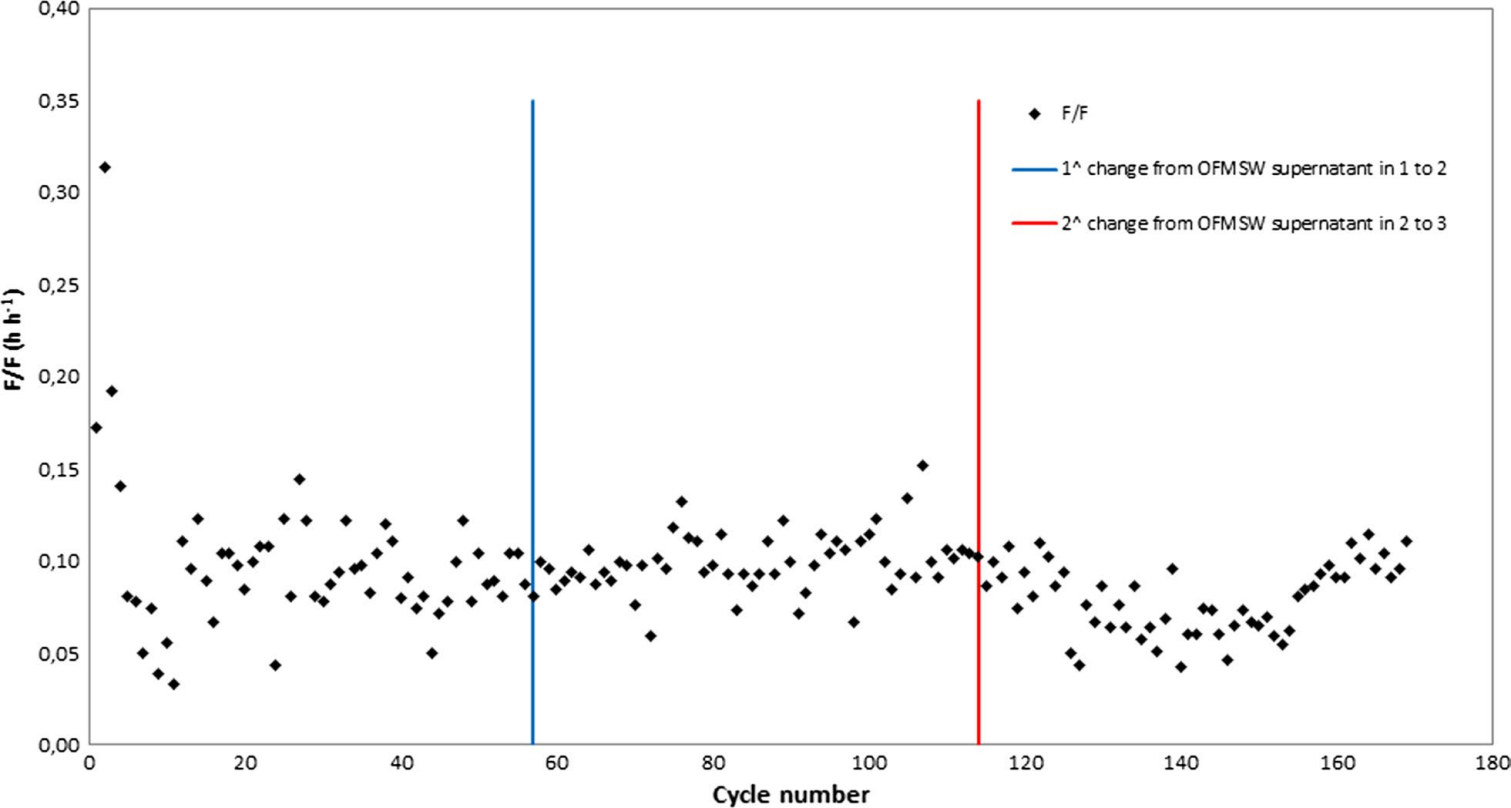
Feast-to-famine ratio (F/F) during the entire selection process with the three OFMSW-supernatants_in_

Feast-to-famine ratio obtained during all selection processes using the three OFMSW-supernatants_in_ (F/F ratio ranged from 0.03 to 0.15) (Fig. 2), confirmed the good performance of the selection procedures, indicating successful enrichment in PHA-accumulating bacteria [23].

### PHA production

Accumulation tests (Table 3) revealed a better performance for the cultures selected with OFMSW-supernatants_in_ 1 and 2 than that of 3 in terms of PHA accumulation in relation to VSS. Maximum PHA content at the end of the tests fed with OFMSW-supernatants_acc._ 1 and 2 was of 467 ± 41 g kg^−1^ VSS and 465 ± 21 g kg^−1^ VSS, respectively, which were about 13% higher than that obtained in the tests fed with OFMSW-supernatant_acc._ 3 (Table 3). These results were similar to those obtained in previous work using other waste substrates (e.g. palm oil mill effluent, fermented olive oil mill pomace, crude glycerol) [31–33], indicating a reliable performance of the cultures in accumulation tests. In terms of PHA storage yields (expressed as COD) on COD consumed, PHA accumulation carried out by using the three supernatants gave substantially similar results when OFMSW-supernatants_acc._ 2 and 3 were used, i.e. 0.5 mg mg^−1^ COD_cons._. These data were higher than those detected for the accumulation trial using OFMSW-supernatant_acc._ 1.

PHA storage yields (expressed as COD) referred to as consumed-OA were very different for different supernatants. For example, PHA yield was reduced from 1.13 to 0.66 mg mg^−1^ COD_OA-cons._ when comparing the trial performed using the OFMSW-supernatant_acc._ 1 to the one using the OFMSW-supernatant_acc._ 3. Residual OA could be potentially reused to produce PHA or destined to the production of biogas and energy, supporting the entire process throughout anaerobic digestion. These solutions have not been considered in this work.

Trials performed using the OFMSW-supernatants_acc._ 1 and 2, gave the highest PHA production with respect to the total COD and OA fed, i.e., as average, 130 ± 16 g kg^−1^ COD_in_ and 223 ± 28 g kg^−1^ OA_in_, that were 30% higher than data obtained for the culture fed with the OFMSW-supernatant_acc._ 3. Lower PHA production was probably due to the decrease of biomass which occurred in the SBR during the last month (data not reported).

PHA composition was constant among the three different accumulation tests performed, with an average value of 56 ± 3% of HB and 44 ± 3% of HV, as detected by the GC–MS approach. This composition was similar to that obtained by Amulya et al. in 2015 [17] using fermented OFMSW as substrate.

## Discussion

### PHA production and mass balance

Results above discussed indicated that supernatants obtained worked well as substrates to select and enrich MMC in PHA-storing bacteria. In particular, PHA content at the end of the feast phase and PHA yield on COD and on OA consumed obtained in this work were comparable or higher than those reported in the literature for selection processes of mixed microbial cultures obtained by feeding similar OFMSW leachate [17, 18] or other waste substrates (e.g. fermented molasses and fermented cheese whey) [10, 29]. Best performances obtained could be due to the fact that in this work culture enrichment was performed at 30 °C, which is an optimal temperature for microbial activity, and because a more balanced C:N ratio (close to 10) was adopted with respect to the lower values adopted by other authors [15, 18].

The good results above discussed allowed us to get PHA contents during PHA accumulation tests higher than those reported in the literature by Amulya et al. in 2015 [17] and comparable to the data reported by Zhang et al. in 2014 [16], both working on food wastes (Table 4). On the other hand, PHA contents were clearly lower than those obtained by Korkakaki et al. in 2016 [18], who obtained PHA contents of 778–784 g kg^−1^ VSS (Table 4) by feeding fermented food wastes to a MMC, achieving very similar results to those reported for pure cultures (860–870 g kg^−1^ VSS by using *Cupriavidus necator*) [34, 35] (Table 4). Nevertheless, it should be specified that those data were obtained by using a mix of synthetic volatile fatty acids (VFAs) (75–90% w/w) and OFMSW leachate (10–25% w/w) during the selection procedure. Korkakai et al. in 2016 reported that the use of pure leachate as the sole substrate to select PHA-accumulating bacteria was less successful than the use of a feed made mainly by synthetic VFAs, because the microbial culture selected with VFAs maintained a high PHA storage capacity even if fed with pure leachate.

**Table 4:** Comparison between studies related to PHA production using fermented OFMSW as substrate and this study

Culture	Substrate	Organic acids composition^a^ (%, weight basis, acetate/n-butyrate/propionate/valerate/isobutyrate/lactate)	PHA content^b^ (g kg^−1^ VSS)	Polymer composition PHB/PHA (w/w)	PHA yield^c^ (mg mg^−1^ COD_cons._)	PHA yield^d^ (mg mg^−1^ COD_OA-cons._)	PHA yield^e^ (g kg^−1^ OFMSW_TS_)	References
MMC	Fermented OFMSW	30/0/70/0/0/0	600	0.77	–	–	25	[15]
MMC	Fermented food waste and sewage sludge	–	477	0.38	–	0.54	–	[16]
MMC	Fermented OFMSW	51.6/22.8/21.1/1.8/2.8/0	237 ± 1	0.61	0.17	–	–	[17]
MMC	Leachate	–	784	–	–	–	–	[18]^f^
MMC	Leachate	–	778	–	–	–	–	[18]^g^
MMC	Percolate 1	39.8/15.3/40.3/0/0/4.6	476 ± 41	0.57 ± 0.04	0.44 ± 0.17	1.13 ± 0.18	62.9 ± 10.5	This study
MMC	Percolate 2	37.1/10.2/46.1/2.7/2.2/1.7	465 ± 21	0.54 ± 0.02	0.52 ± 0.10	0.75 ± 0.16	114 ± 14	This study
MMC	Percolate 3	45/20/29.4/2.8/1.1/1.7	405 ± 28	0.57 ± 0.01	0.50 ± 0.10	0.65 ± 0.06	100 ± 6	This study
*Cupriavidus necator* CCGUG 52238	Fermented food waste	6/0/0/0/0/94	860	1	–	–	–	[34]
*Cupriavidus necator H16*	Fermented food waste	–	870	1	–	–	–	[35]

^a^See Additional file 1

^b^PHA accumulated at the end of the accumulation test referred to VSS

^c^PHA storage yield expressed as COD (COD_PHA_) referred to COD_cons._

^d^PHA storage yield expressed as COD (COD_PHA_) referred to COD_OA-cons._

^e^PHA produced referred to OFMSW_TS_

^f^Accumulation test with a biomass selected with a substrate made of 90% synthetic VFAs and 10% pre-treated leachate

^g^Accumulation test with a biomass selected with a substrate made of 75% synthetic VFAs and 25% pre-treated leachate

The PHA yields obtained, i.e. 0.44–0.52 mg mg^−1^ COD_cons._ and 0.65–1.13 mg mg^−1^ COD_OA-cons._ (Table 4), were very good and comparable to those reported in the literature by using other waste substrates to produce PHA (e.g. fermented olive oil mill effluent, fermented molasses, fermented cheese whey) [10–12]. Moreover, results from this work were higher than that reported by Zhang et al. in 2014 [16] concerning the use of fermented food waste and sewage sludge as the substrate for PHA production (Table 4).

Table 5 reports the mass balance of the entire process: the percolation process producing OA (first stage) plus PHA accumulation (second stage).

**Table 5:** PHA yield on OFMSW weight unit

	First stage		Second stage	Global process	
	Percolate produced (L kg^−1^ OFMSW_w.w._)	OA produced^a^ (g kg^−1^ OFMSW_w.w._)	PHA yield^b^ (g kg^−1^ OA_in_)	PHA yield^c^ (g kg^−1^ OFMSW_TS_)	PHA yield^d^ (g kg^−1^ OFMSW_w.w._)
Trial 1	8.03	60.5	230 ± 38	62.9 ± 10.5	13.9 ± 2.3
Trial 2	19.3	151	220 ± 28	114 ± 14	33.2 ± 4.2
Trial 3	25.6	139	161 ± 9	100 ± 6	22.4 ± 1.3

^a^OA expressed as acetic acid, determined according to the acid titration method [27]

^b^PHA produced referred to OA_in_

^c^PHA produced referred to OFMSW_TS_

^d^PHA produced referred to OFMSW_w.w_

Percolation trials indicated that the process parameters adopted for Trial 2 determined the highest percolate and OA production per kg of OFMSW treated, giving the best trial results among the three tested. This evidence together with the very good performance in converting OA into PHA (second stage) using the OFMSW-supernatant_acc._ 2, resulted in the highest PHA production among the three two-stage processes, i.e. 33.22 ± 4.2 g kg^−1^ of OFMSW_w.w._, corresponding to 114.4 ± 14.5 g kg^−1^ of OFMSW_TS_, almost 5 times higher than that reported by Rhu et al. in 2003 [15] (25 g of PHA kg^−1^ of dry food waste).

### PHA characterization

Mass balance previously discussed indicated that Trial 2 gave the best results in terms of total PHA yield. Therefore, PHA characterization was focused on the products obtained in Trial 2.

**Fig. 3:**
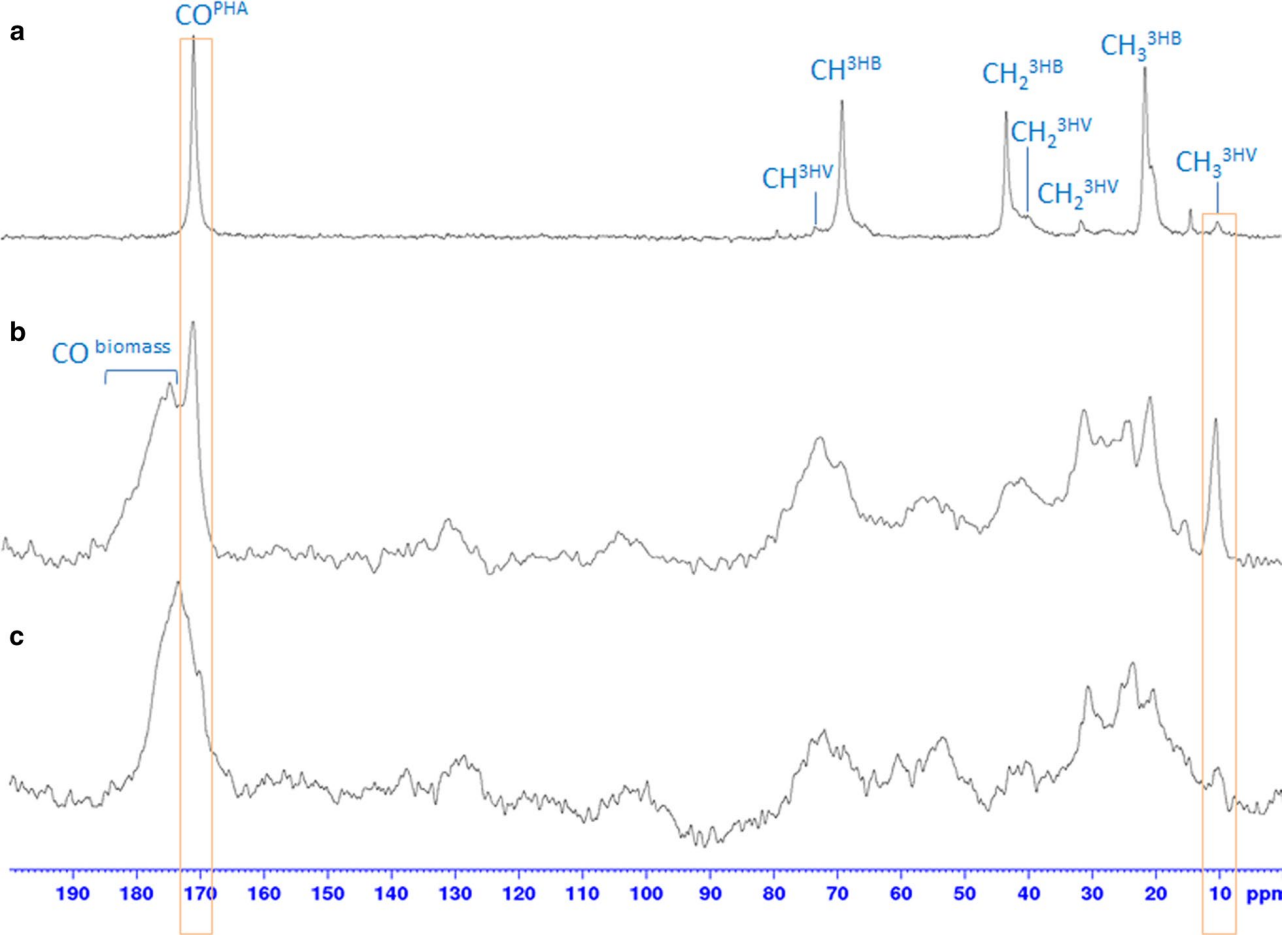
^13^C SPE NMR spectra of the reference pure PHA^88/12^ (**a**), original biomass containing PHA (**b**) and exhausted biomass after PHA extraction (**c**). *Frames* indicate CO and CH_3_3HV signals related to PHA polymer that can be used for monitoring the synthesis and extraction process

Solid-state ^13^C NMR was used to evaluate the content of PHA in the generated biomass and to monitor the PHA extraction process. ^13^C cross polarization NMR technique, based on the carbon magnetization transferred from ^1^H protons, could not be used for quantification of different chemical species/functional groups. In contrast, single-pulse excitation (SPE) NMR is based on the direct ^13^C nuclei polarization and, even if less sensitive than CP MAS NMR, can be optimized for quantitative evaluation analysis. The most critical parameter, recycle delay, was set at 160 s, in order to cover T1 values of each ^13^C type [36]. The obtained ^13^C SPE NMR of the reference PHA standard, biomass enriched in PHA and the biomass after PHA extraction are shown in Fig. 3.

Together with the PHA signals, the biomass spectrum contains signals compatible with proteins (large CO peak at 170–185 ppm), minor polysaccharides (large peak of anomeric O–C–O at 100–110 ppm), etc. It is worth noting that PHA-related peaks are sharper than the other signals, indicating that these polymers were present, at least partially, in their semi-crystalline form.

In spite of the optimized acquisition parameters, quantitative analysis of PHA in biomass was limited by strong signal overlapping, especially in the aliphatic region (0–80 ppm) (Fig. 3). As a tentative for PHA quantitative evaluation in the original biomass, a ratio between CO^PHA^ and CO^biomass^ was estimated as 30–35 molar %, using a Topspin software for peak integral simulation. Since the result significantly depends on the estimated signal width, such an approach could not be applied for the exhausted biomass, where the CO^PHA^ peak is not sufficiently high. It still can be clearly observed from the decreased signals of CO^PHA^ (~172 ppm) (Fig. 3) and well isolated CH_3_^3HV^ (~10 ppm) (Fig. 3) that the biomass treated with chloroform contained PHA in lower quantity. Notably, even before extraction, it can be seen that the synthesized PHA are characterized by higher content of 3HV monomer that is in agreement with GC–MS data. Altogether, these data can be useful for monitoring the PHA content in biomass during their production and extraction.

**Fig. 4:**
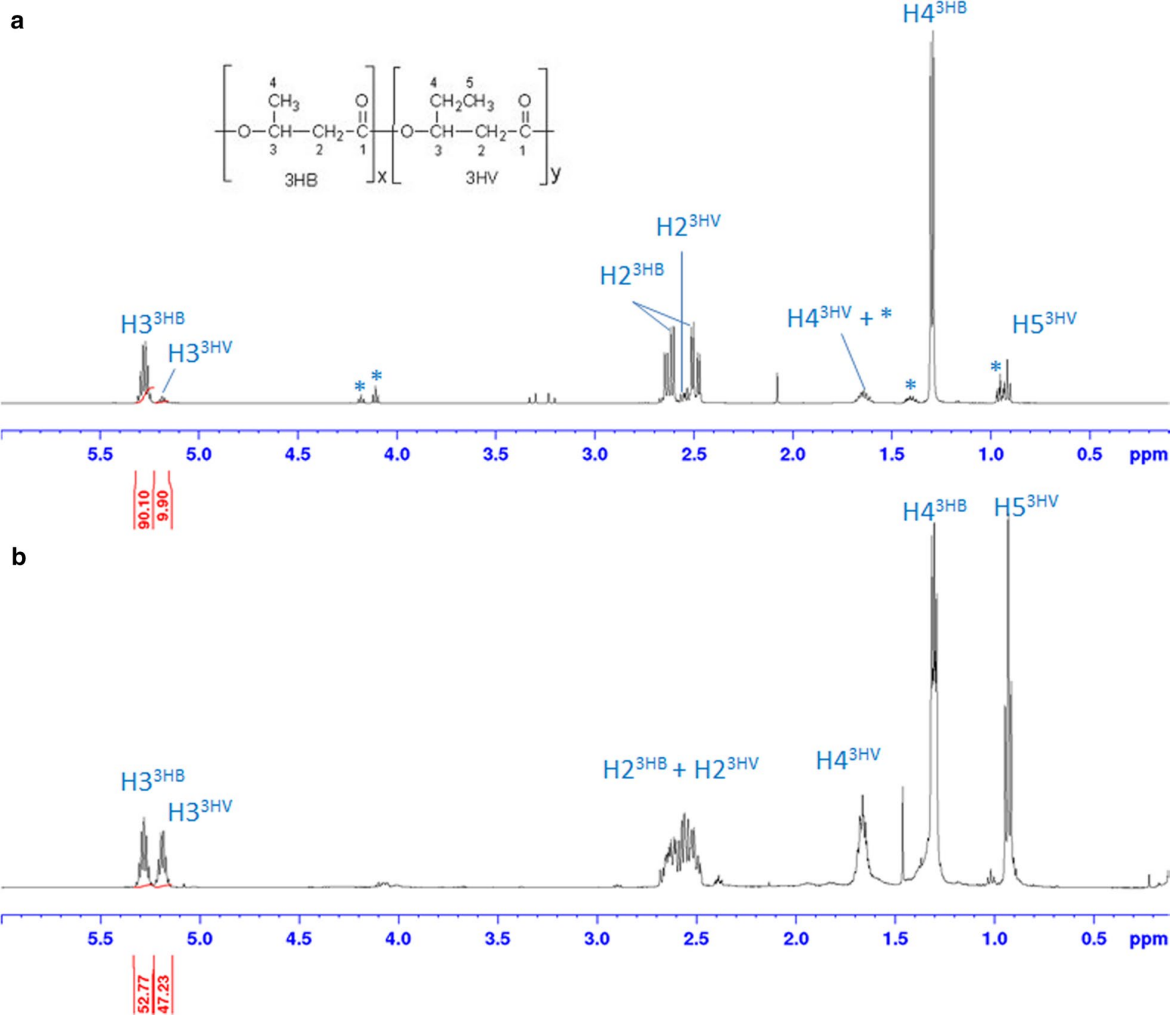
^1^H NMR spectra of the reference PHA^88/12^ (**a**) and the extracted PHA (**b**) in CDCl_3_. *Asterisks* indicate the signals related to impurities present in commercial PHA sample

In order to provide more detailed structural features of the extracted PHA polymers, solution ^1^H NMR, GC–MS and GPC-TDA were applied along with solid-state NMR. By doing so, extracted PHA was characterized by ^1^H NMR using standard PHA^88/12^ for signal assignment (Fig. 4).

The PHA-related signals assigned using ^1^H and COSY NMR (*not shown*) were in agreement with the previously published data [37]. The molar 3HB/3HV composition for commercial standard PHA^88/12^ and biosynthesized PHA determined by ^1^H NMR is reported in Table 6. Notably, quantitative data obtained for the biosynthesized PHA by ^1^H NMR (53/47%) (Table 6) are also in agreement with GC–MS results.

**Table 6:** Characterization of PHA produced in the accumulation stage of Trial 2 compared with commercial PHA^88/12^

Products	HB/HV^a^ (molar ratio)	Mw^b^ (kDa)	Polydispersity	Rh^c^ (nm)	*a* ^d^	log*K* ^d^
Commercial reference—PHA^88/12^	90/10	2∙10^5^	1.3	14	0.70	−3.8
Extracted PHA from Trial 2	53/47	8∙10^5^	1.4	29	0.65	−3.4

^a^Molar ratio such as detected by NMR

^b^Molecular weight distribution determined by GPC-TDA

^c^Hydrodynamic Radius determined by GPC-TDA

^d^Mark–Houwink parameters *a* and log*K*

Gel permeation chromatography with a TDA detection system was used to describe the molecular weight distribution of the biosynthesized PHA. Separation and detection conditions (including *dn/dc* calculation) were optimized using a commercial PHA^88/12^ sample. Table 6 reports the average molecular weight Mw, polydispersity and hydrodynamic radius Rh determined for the reference PHA^88/12^ and extracted PHA sample. In spite of a similar polydispersity, the biosynthesized PHA sample was characterized by higher molecular weight and hydrodynamic radius. Mark–Houwink parameters *a* and log*K*, reflecting conformational behaviour of polymers in solution, are also reported in Table 6. The obtained *a* values for reference PHA^88/12^ and extracted PHA are compatible with the values characteristic for flexible polymers in solution. Interestingly, these properties do not depend on the differences in molecular weight and chemical composition between the two samples.

## Conclusions

Data obtained in this work can be useful as the starting point for considering both technical and economic feasibility of PHA production from OFMSW by using MMC.

The optimization of OFMSW acidic fermentation resulted a key step for the success of PHA production because it allows the production of a large amount of OA to be used to produce PHA. Moreover, the high concentrations of OA acted to prevent biogas (methane) production from consuming the substrate to be used to produce PHA. This kind of process could be rapidly implemented in a full-scale plant by adapting dry anaerobic digestion that is commonly used to produce biogas from household wastes.

This work, for the first time, reported that PHA yield referred to an OFMSW weight unit in an optimized process. Therefore, taking into consideration a PHA value of 2600–5800 $ tonne^−1^ [38], data of total food waste produced in EU (87.6 million tonnes) and PHA yield obtained in this work (33.2 g kg^−1^ OFMSW_w.w._), in theory a total gross revenue of 7.6–16.9 billion $ might be achieved.

## Additional file

**Additional file 1.** Specific acids composition of fermented OFMSW used as substrate for PHA production in literature and in this study. Table reporting the specific acids composition of fermented OFMSW used as substrate for PHA production in literature and in this study.

## Abbreviations

OA: organic acids
OFMSW: organic fraction of municipal solid waste
PHA: polyhydroxyalkanoates
FW: food waste
MMC: mixed microbial cultures
APBR: anaerobic percolation biocell reactor
SBR: sequencing batch reactor
OFMSW-supernatant_in_: substrate for PHA selection phase
OFMSW-supernatants_acc._: substrate for PHA accumulation phase
W.W.: wet weight
TS: total solids
VS: volatile solids
TSS: total suspended solids
VSS: volatile suspended solids
COD_PHA_: PHA expressed as COD
COD_in_: COD fed
COD_cons._: COD consumed
OA_in_: organic acids fed
COD_OA-cons._: organic acids consumed expressed as COD
X: active biomass
COD_X_: active biomass expressed as COD
HB: hydroxybutyrate
HV: hydroxyvalerate
