# Supplementary material for: Enhanced polyhydroxyalkanoate (PHA) production from the organic fraction of municipal solid waste by using mixed microbial culture
Source: Biotechnol Biofuels. 2017 Aug 22;10:201. doi: 10.1186/s13068-017-0888-8 (PMC5567430; doi:10.1186/s13068-017-0888-8)
Supplement: Supplementary file 1 — Additional file 1. Specific acids composition of fermented OFMSW used as substrate for PHA production in literature and in this study. Table reporting the specific acids composition of fermented OFMSW used as substrate for PHA production in literature and in this study. [file 13068_2017_888_MOESM1_ESM.pdf]

Additional file 1

Specific acids composition of fermented OFMSW used as substrate for PHA production in literature and in this study

Substrate	Acetate (%, weight basis on total OA)	n-butyrate (%, weight basis on total OA)	Propionate (%, weight basis on total OA)	Valerate (%, weight basis on total OA)	Isobutyrate (%, weight basis on total OA)	Lactate (%, weight basis on total OA)	References
**Fermented OFMSW**	30	0	70	0	0	0	15
**Fermented OFMSW**	51.6	22.8	21.1	1.8	2.8	0	17
**Percolate 1**	39.8	15.3	40.3	0	0	4.6	This study
**Percolate 2**	37.1	10.2	46.1	2.7	2.2	1.7	This study
**Percolate 3**	45	20	29.4	2.8	1.1	1.7	This study
**Fermented food waste**	6	0	0	0	0	94	34
